# A process for analysis of microarray comparative genomics hybridisation studies for bacterial genomes

**DOI:** 10.1186/1471-2164-9-53

**Published:** 2008-01-29

**Authors:** Ben Carter, Guanghui Wu, Martin J Woodward, Muna F Anjum

**Affiliations:** 1 Department of Food and Environmental Safety, Veterinary Laboratories Agency-Weybridge, New Haw, Addlestone, Surrey KT15 3NB, UK; 2 South East Wales Trials Unit, Dept. Primary Care and Public Health, School of Medicine, Heath Park, Cardiff University, CF14 4XN, UK

## Abstract

**Background:**

Microarray based comparative genomic hybridisation (CGH) experiments have been used to study numerous biological problems including understanding genome plasticity in pathogenic bacteria. Typically such experiments produce large data sets that are difficult for biologists to handle. Although there are some programmes available for interpretation of bacterial transcriptomics data and CGH microarray data for looking at genetic stability in oncogenes, there are none specifically to understand the mosaic nature of bacterial genomes. Consequently a bottle neck still persists in accurate processing and mathematical analysis of these data. To address this shortfall we have produced a simple and robust CGH microarray data analysis process that may be automated in the future to understand bacterial genomic diversity.

**Results:**

The process involves five steps: cleaning, normalisation, estimating gene presence and absence or divergence, validation, and analysis of data from test against three reference strains simultaneously. Each stage of the process is described and we have compared a number of methods available for characterising bacterial genomic diversity, for calculating the cut-off between gene presence and absence or divergence, and shown that a simple dynamic approach using a kernel density estimator performed better than both established, as well as a more sophisticated mixture modelling technique. We have also shown that current methods commonly used for CGH microarray analysis in tumour and cancer cell lines are not appropriate for analysing our data.

**Conclusion:**

After carrying out the analysis and validation for three sequenced *Escherichia coli *strains, CGH microarray data from 19 *E. coli *O157 pathogenic test strains were used to demonstrate the benefits of applying this simple and robust process to CGH microarray studies using bacterial genomes.

## Background

DNA microarray technologies enable comparison of the genetic composition in a variety of organisms starting from mosaic bacterial genomes to chromosomal aberration in cancer cells. Arrays are produced by printing discrete regions of the genome such as open reading frames, short oligonucleotide probes or whole genomes tiled onto a glass slide or nitrocellulose substrate in an ordered array of spots. Each spot acts as a device to determine if the same region is conserved or stable in the genome of test and reference strains. This is determined by labelling genomic DNA from the test and reference sample with different fluorescent dyes followed by co-hybridisation to the microarray slide. If a gene is conserved in both samples the fluorescence emitted at the corresponding position on the array will be a mixture derived from both labels. If the gene is present in only one sample then only one type of fluorescence will be observed. The use of a reference sample in co-hybridisation experiments internally controls for defects in slide printing and hybridisation [1].

Comparative Genomic Hybridisation (CGH) microarray studies are being used increasingly to look at alterations in chromosomal DNA in a wide variety of circumstances. This ranges from studies looking at genome aberrations in cancer or tumour cells to genome diversity in bacterial cells. Several studies have shown that during the development and progression of various cancers amplification, deletion or translocation of chromosomal segments occurs resulting in the malfunctioning of cellular processes. Comparison of the log-ratios intensities from CGH microarray data from diseased versus control samples has been used widely to measure such changes [2-5]. Indeed, several algorithms and software has been developed to identify such aberrations within the chromosome, with the goal being to identify regions of concentrated high and low log-ratios [6-9]. These software methods can be broadly categorised into smoothing or segmentation algorithms. The smoothing algorithms use information from a number of genes locally to assign the log_2_(Cy3/Cy5), whereas the segmentation algorithms define the set of genes. It has been shown when there are many smaller regions with little consistency of log_2_(Cy3/Cy5) neither of these algorithms may be effective [10].

In bacteria gene duplication and rearrangements occurs often and randomly throughout the chromosome [11,12]. However, the major driving force for bacterial evolution is horizontal gene transfer (HGT) whereby integrating viruses (phages), transposons and other mobile elements are inserted within the host bacterial genome. The elements are flanked by direct nucleotide repeats and often inserted in the vicinity of tRNAs. Insertion and deletion of these elements are common with repeated events of gene acquisition and loss resulting in a highly variable gene content [13-18]. Therefore a major aim of bacterial CGH microarray study has been to assess the plasticity of bacterial genome structures both within and between species (or subspecies) to deduce the evolutionary relatedness of bacterial pathogens such as *Salmonella *[19-21], or to understand the genetic diversity of field and clinical isolates within bacterial species such as *Escherichia coli*, *Shigella, Mycobacterium*, and *Staphylococcus*, with respect to a representative sequenced strain [22-25].

In bacterial CGH studies, the fluorescent signal intensity is used to estimate which genes are conserved or variable in unsequenced strains. However, one of the critical problems faced in interpreting microarray data from bacterial CGH studies where DNA hybridisation results from unsequenced strains of field and clinical origin are compared to a control sequenced strain, is determining those genes that are conserved between and within species, from divergent or highly polymorphic genes (such as those encoding the flagellar sub-unit, O-lipopolysaccharide, verotoxin, and intimin, amongst others [26-28]), and absent genes. A conserved gene is hypothesised to have approximately equal signal intensity in the test (Cy3) and control (Cy5) channels, whilst a divergent or absent gene has a true signal in the control channel only. Thus, the genome content of bacterial strains of unknown origin can be estimated by CGH microarrays, where a cut-off algorithm determines the position of separation between genes present and those divergent or absent. Hybridisations with less bound material on the test channel than the determined cut-off are categorised as divergent or absent, those with higher signals are referred to as present. Here we compare the naïve cut-off with other more dynamically derived cut-off algorithms and establish a robust process for analysing bacterial CGH microarray data, using strains selected from the pathogenic *E. coli *O157 serotype and an *E. coli *K12 laboratory strain, that may be automated in the future. A unique point in our study was the inclusion of genomic DNA from three bacterial genomes in the control channel, to provide a baseline for all genes present in the *E. coli *O157 panarray used in this study.

## Results

### Process for the analysis of bacterial CGH data

**Figure 1:**
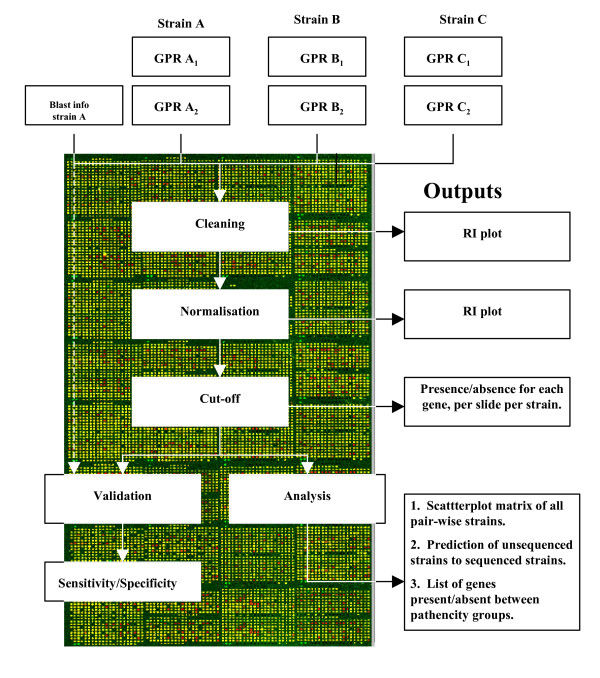
**The analysis process for CGH studies**. The minimum number of stages that are required to carry out a CGH study from raw data to a validated output and have confidence in the robustness of the results, are outlined. Stages include data cleaning, normalisation and decision over the presence/divergence of each gene in the array. The validation provides a metric to compare the process by examining sequenced data.

Microarray hybridisations were performed on three *E. coli *sequenced strains and 19 *E. coli *O157 test strains against the three sequenced strains, as described in Materials and Methods. These provided scanned images that were converted into signal and background intensity values for both the Cy3 and Cy5 channels, for each spot on each slide. The log_2_(Cy3/Cy5) was cleaned and normalised as described in the Methods section. The data was then analysed using different cut-off algorithms described below. The process used for analysis of sequenced reference and unsequenced test strains from CGH microarray studies are shown in Figure 1.

#### Validation

Within the framework outlined in Figure 1 the validation was carried out on microarray hybridisation datasets from three *E. coli *sequenced strains (MG1655, EDL933 and Sakai) using each of the cut-off methods described below. This resulted in a number of genes to be identified as present and absent or divergent. The validation step reported the number of correctly or falsely identified present and absent genes after comparing the data with BLASTN data for the three sequenced strains.

#### Comparison of the cut-off algorithms

**Figure 2:**
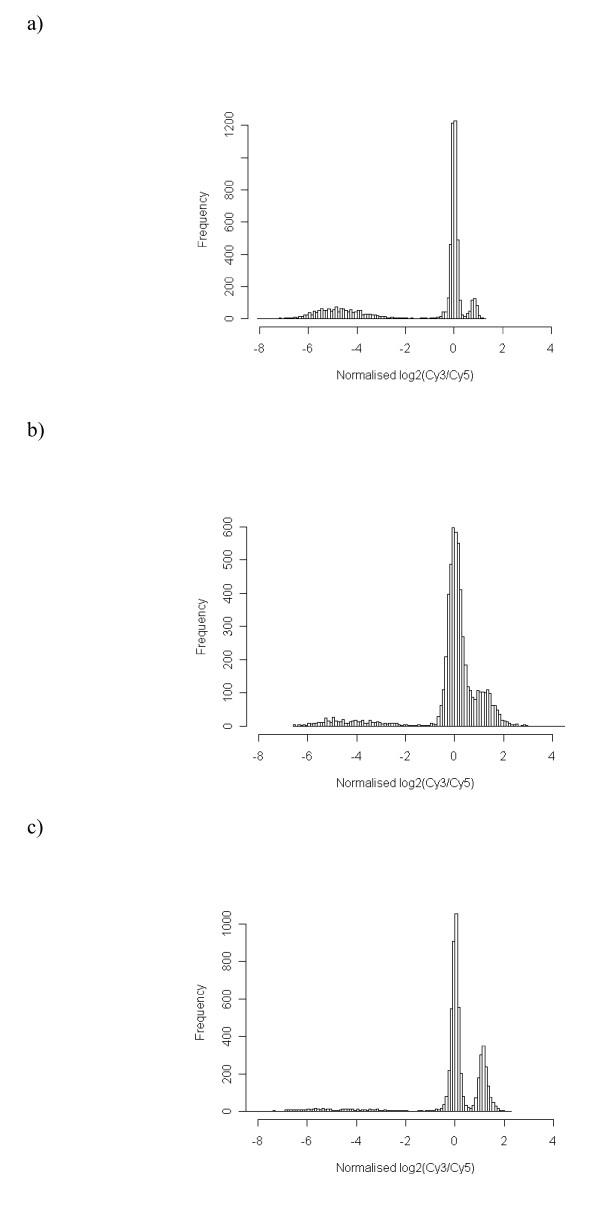
**Distribution of the log_2_(Cy3/Cy5) data for each sequenced strain represented as a histogram**. a) Distribution of log_2_(Cy3/Cy5) data for MG1655. b) Distribution of log_2_(Cy3/Cy5) data for EDL933. c) Distribution of log_2_(Cy3/Cy5) data for Sakai.

Each algorithm calculated a cut-off using data from each slide with the exception of the naïve cut-off. Using the cut-off, including various naïve cut-off scores, and BLASTN data, the sensitivity, specificity and the M-Score (a method for combining both the sensitivity and specificity [29]) were calculated, weighted by prevalence, and validated the accuracy of each algorithm. In this application, the prevalence of the conserved genes was different between strains, so sensitivity was weighted by the conserved gene prevalence. The M-Score allowed the combination of both summary statistics, in a manner that accounts for strains to have different proportions of conserved genes. Figures 2 present histograms of the log_2_(Cy3/Cy5) data for the three sequenced strains, and the summary statistics calculated from the algorithms are given in Tables 1 to 3.

**Table 1:** Comparison of the six algorithms, using the microarray hybridization data from the MG1655 sequenced strain. The result of using each algorithm were compared to BLASTN data and are shown below.

	Cut-off	TP	FP	TN	FN	Sensitivity	Specificity	M-Score
	0.50	4231	3	1452	58	98.65	99.78	98.90
	0.33	4244	6	1449	45	98.95	99.59	99.11
Naïve	0.25	4249	10	1445	40	99.07	99.31	99.12
Cut-off	0.20	4253	20	1435	36	99.16	98.63	99.02
	0.10	4265	125	1330	24	99.44	91.40	97.41

GENCOM*		4203	3	1452	86	97.99	99.79	98.45

GACK**	EPP = 50	4116	1	1554	173	95.97	99.93	96.97
	EPP= 0	4202	3	1452	87	97.97	99.79	98.43

Porwollik		4219	2	1453	70	98.37	99.86	98.75

MKD***		4243	12	1443	46	98.93	99.18	98.99

Mixture	Bimodal	4256	20	1435	33	99.23	98.63	99.07
	Trimodal	4236	4	1451	53	98.76	99.73	99.01

The number of genes estimated as correctly conserved (True positives, TP), genes identified as conserved but actually are variable (false positives, FP), genes identified as correctly variable (true negatives TN), and genes identified falsely as variable (false negative FN), are given. The sensitivity, specificity, and M-Score are also calculated, where the sensitivity = TP/(TP+FP), specificity = TN/(FP+TN), and M-Score = Sensitivity*prevalence + Specificity*(1-prevalence)

* Institute of Food Research method (GENCOM)

** Genotyping Analysis by Charlie Kim method (GACK)

*** Minimum Kernel Density method (MKD)

**Table 2:** Comparison of the six algorithms, using the microarray hybridization data from the EDL933 sequenced strain. The result of using each algorithm were compared to BLASTN data and are shown below.

	Cut-off	TP	FP	TN	FN	Sensitivity	Specificity	M-Score
	0.50	5202	24	475	43	99.18	95.19	98.83
Naïve	0.33	5211	31	468	34	99.35	93.79	98.87
cut-off	0.25	5218	37	462	27	99.49	92.59	98.89
	0.20	5222	43	456	23	99.56	91.38	98.58
	0.10	5235	127	372	10	99.81	74.55	97.61

GENCOM*		5167	21	478	78	98.31	95.79	98.09

GACK**	EPP = 50	5083	22	477	162	96.91	95.59	96.80
	EPP= 0	5197	24	475	48	99.08	95.19	98.75

Prowollik		5185	23	476	57	98.91	95.39	98.60

MKD***		5207	29	470	38	99.28	94.18	98.84

Mixture	Bimodal	5215	37	462	30	99.42	92.59	98.83
	Trimodal	5207	27	472	38	99.27	94.59	98.86

The number of genes estimated as correctly conserved (True positives, TP), genes identified as conserved but actually are variable (false positives, FP), genes identified as correctly variable (true negatives TN), and genes identified falsely as variable (false negative FN), are given. The sensitivity, specificity, and M-Score are also calculated, where the sensitivity = TP/(TP+FP), specificity = TN/(FP+TN), and M-Score = Sensitivity*prevalence + Specificity*(1-prevalence)

* Institute of Food Research method (GENCOM)

** Genotyping Analysis by Charlie Kim method (GACK)

*** Minimum Kernel Density method (MKD)

**Table 3:** Comparison of the six algorithms, using the microarray hybridization data from the Sakai sequenced strain. The result of using each algorithm were compared to BLASTN data and are shown below.

	Cut-off	TP	FP	TN	FN	Sensitivity	Specificity	M-Score
	0.50	5285	2	416	41	99.23	99.52	99.25
Naïve	0.33	5297	6	412	29	99.45	98.56	99.39
cut-off	0.25	5302	8	410	24	99.55	98.09	99.44
	0.20	5308	12	406	18	99.66	97.13	99.48
	0.10	5316	64	354	10	99.81	84.69	98.71

GENCOM*		5238	2	416	88	98.34	99.52	98.43

GACK**	EPP = 50	5137	1	417	189	96.45	99.76	96.69
	EPP= 0	5261	1	417	65	98.78	99.76	98.85

Prowollik		5277	1	417	49	99.07	98.76	99.13

MKD***		5297	6	412	29	99.45	98.56	99.39

Mixture	Bimodal	5296	8	413	30	99.44	98.80	99.39
	Trimodal	5244	1	417	82	98.46	99.76	98.55

The number of genes estimated as correctly conserved (True positives, TP), genes identified as conserved but actually are variable (false positives, FP), genes identified as correctly variable (true negatives TN), and genes identified falsely as variable (false negative FN), are given. The sensitivity, specificity, and M-Score are also calculated, where the sensitivity = TP/(TP+FP), specificity = TN/(FP+TN), and M-Score = Sensitivity*prevalence + Specificity*(1-prevalence)

* Institute of Food Research method (GENCOM)

** Genotyping Analysis by Charlie Kim method (GACK)

*** Minimum Kernel Density method (MKD)

#### Assessment of the K-12 (MG1655) data

The normalised data were visualised prior to applying the different cut-off algorithms (Fig. 2a). Three well defined distributions were obtained. The data was focused on the primary mode centred about log_2_(Cy3/Cy5) = 0, these genes were expected to be present in both test (MG1655) and control strains (EDL933, Sakai, MG1655). There was a minor secondary mode located to the right of the primary mode that also represented present genes. This feature was due to those genes that are not present in all control strains or when multiple copies of genes were present in the test strain, therefore, generating an elevated log_2_(Cy3/Cy5).

The cut-off algorithms were then applied to the normalised data (for the GENCOM and Porwollik methods unnormalised Cy3 and Cy5 data was used). From Table 1, the naïve cut-off was optimised at 0.25, with a sensitivity of 99.07 (min = 98.65, max = 99.44), and specificity of 99.31 (min = 91.40, max = 99.77). Therefore, although the naïve cut-off performed well due to the clearly defined modes and little replication error, the algorithm may perform poorly under different conditions when a range of modes have not been considered [30].

**Figure 3:**
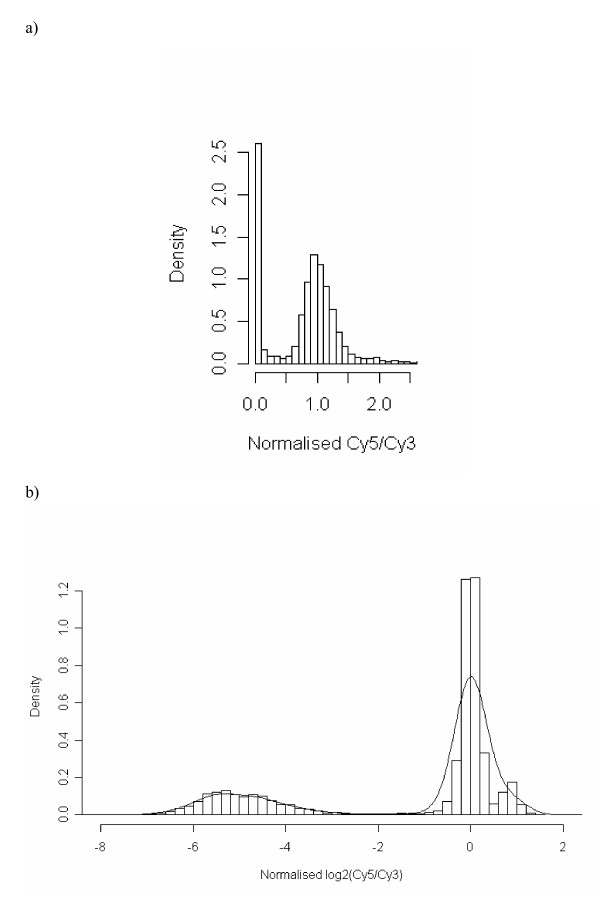
**Histogram of MG1655 hybridisation data**. A histogram of the MG1655 microarray hybridisation data is shown (slide number 12842588). The data is displayed the raw scale (a) and on the log_2 _scale, with the scaled kernel density superimposed (b).

The naïve cut-off at 0.25 was shown in Table 4 to present the highest M-Score, with the mixture model the second highest (sensitivity = 99.23, and specificity = 98.63), and Minimum Kernel Density (MKD) the third highest (sensitivity = 98.99, and specificity = 98.37). The ranked order of the algorithms in Table 4 suggested that the naive cut-off at 0.25 was the best approach due to the highest M-Score. Therefore, the optimal cut-off was data dependent and was empirically derived. Hence, the disadvantage of this method is that it cannot be automated unlike the other approaches and a range of cut-off values needs to be considered to derive at the optimal value. The approach with the second highest M-Score was the mixture model. However, due to the complexity of fitting mixture model the simpler MKD approach was considered more appropriate. An example of an out put from this algorithm has been presented for normalised MG1655 data within Figure 3, and shows the simplicity of this approach.

**Table 4:** Summary of the performance of each algorithm using CGH microarray data for the MG1655 sequenced strain. The sensitivity, specificity, and M-score generated from each of the cut-off algorithms from the CGH data were summarized for comparison.

Algorithm	M-Score	Sensitivity	Specificity
Naive Cut-off (0.25)	99.12	99.07	99.31
Mixture Model (Bimodal)	99.07	99.23	98.63
MKD	98.99	98.93	99.18
Porwollik	98.75	98.37	99.86
GENCOM	98.45	97.99	99.79
GACK EPP = 0	98.43	97.97	99.79

* Institute of Food Research method (GENCOM)

** Genotyping Analysis by Charlie Kim method (GACK)

*** Minimum Kernel Density method (MKD)

#### Assessment of the EDL933 data

The normalised data was visualised before applying the cut-off algorithms (Fig. 2b). The distribution was focused towards a positively skewed primary mode centred about log_2_(Cy3/Cy5) = 0. To the left of the primary mode were far fewer absent genes than seen in the K-12 example (Fig 2a). Therefore, the EDL933 data set was less well separated into three modes compared to K-12 presenting greater difficulty in discerning the present genes from those absent or divergent. So interpretation of data from subsequent cut-off algorithms proved more difficult than for the K-12 data.

Application of cut-off algorithms resulted in the naïve cut-off to be optimised at 0.25, with a sensitivity of 99.49 (min = 99.18, max = 99.81), and specificity of 92.59 (min = 74.55, max = 95.19; Table 2). The range of these results showed that the naïve cut-off was varied and unreliable because of the lower specificity especially at cut off value 0.1.

From Table 5 the naïve cut-off at 0.25 was shown to present the highest M-Score, then the mixture model, with the MKD method the third highest (sensitivity = 99.28, and specificity = 94.18). Although the ranked order of the algorithms in Table 5 suggest the naive cut-off at 0.25 was the best approach, five naïve cut-off values were used and only optimal results at 0.25 displayed. As mentioned already, a range of naïve cut-off values is required to be considered for each data set making the method laborious and not suitable for automation. Therefore the optimal automated method was by mixture modelling. However, given the cost in time and complexity of the mixture method the far simpler MKD approach is recommended as the M-score, sensitivity and specificity for this method was very similar to that of mixture modelling.

**Table 5:** Summary of the performance of each algorithm using CGH microarray data for the EDL933 sequenced strain. The sensitivity, specificity, and M-score generated from each of the cut-off algorithms from the CGH data were summarized for comparison.

Algorithm	M-Score	Sensitivity	Specificity
Naive Cut-off (0.25)	98.89	99.49	92.59
Mixture Model (trimodal)	98.86	99.27	94.59
MKD***	98.84	99.28	94.18
Porwollik	98.60	98.91	95.39
GACK** EPP = 0	98.75	99.08	95.19
GENCOM*	98.09	98.31	95.79

* Institute of Food Research method (GENCOM)

** Genotyping Analysis by Charlie Kim method (GACK)

*** Minimum Kernel Density method (MKD)

#### Assessment of the Sakai data

The normalised data was examined before applying any cut-off algorithms (Fig. 2c). It can be seen that the Sakai data was similar to the EDL933 data (Fig. 2b), but the naïve cut-off was optimised at 0.20, with a sensitivity of 99.66 (min = 99.18, max = 99.81), and specificity of 97.13 (min = 84.69, max = 99.52; Table 3). These showed consistent results for a cut-off above 0.1, but specificity was poor for a cut-off of 0.1.

The naïve cut-off at 0.2 was shown to present the highest M-Score, with the MKD method second highest (sensitivity = 99.45, and specificity = 98.80), again suggesting that the MKD cut-off algorithm was the optimal, as well as the simplest automatic algorithm available (Table 6).

**Table 6:** Summary of the performance of each algorithm using CGH microarray data for the Sakai sequenced strain. The sensitivity, specificity, and M-score generated from each of the cut-off algorithms from the CGH data were summarized for comparison

Algorithm	M-Score	Sensitivity	Specificity
Naive Cut-off (0.20)	99.48	99.66	97.13
MKD***	99.39	99.45	98.80
Mixture Model (trimodal)	99.39	99.44	98.80
Porwollik	99.13	99.07	98.76
GACK** EPP = 0	98.85	98.78	99.76
GENCOM*	98.43	98.34	99.52

* Institute of Food Research method (GENCOM)

** Genotyping Analysis by Charlie Kim method (GACK)

*** Minimum Kernel Density method (MKD)

#### The unsequenced field isolates

We have validated the proposed process using sequenced reference strains. In practice, we wish to compare the presence and absence or divergence of genes in unsequenced strains with respect to sequenced reference strains. We performed forty- four hybridisations on nineteen *E. coli *O157 test isolates, whereby genomic DNA was extracted, microarray hybridisations performed and analysed using the processes described in Figure 1 (see Materials and Methods).

**Figure 4:**
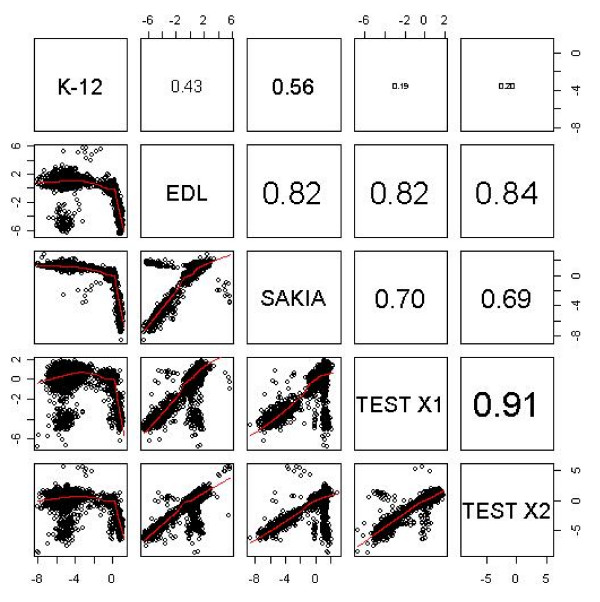
**A scatter plot matrix of unknown test strains**. A scatter plot matrix of the three reference and two test strains (X1, and X2 represent strains 0864/00 and 0330/01) were compared to identify test strains most similar to each sequenced strain. The lower left panes present the scatter plots with smoothing splines and the right hand panel displays the Pearson's correlation coefficient.

The first output of the analysis step for unknown test strains includes performing a scatter plot matrix of all test strains in a pair-wise manner to control strains to analyse the extent of diversity between unknown test strains and the sequenced control strains. An example of this is shown in Figure 4, which shows the extent of diversity of *E. coli *O157 strains 0864/00 (X1) and 0330/01 (X2) to the three sequenced control strains. The matrix of scatter plots (lower left hand panels) and the Pearson's correlation coefficient (upper right hand panel) between the three sequenced and two test strains is shown. Both strains show a higher correlation to EDL933 (X1 = 0.82; X2 = 0.84) than Sakai or MG1655.

In a similar manner Pearson's Correlation co-efficient was used to correlate the remaining unknown *E. coli *O157 strains to the sequenced reference strains (Table 7). The data shows that the genomic composition of the majority of test strains, i.e. seventeen of the nineteen strains used in the study, were more similar to EDL933 than Sakai or MG1655. The range of correlation found between these strains represent typical variability found between clonal isolates, indicating these strains to be closely related [31]. The genomic composition of one O157 isolate (1176/00) was more similar to MG1655 than either O157 sequenced strains, and one isolate (1070/00) showed genomic correlation with Sakai, although the value was relatively low in comparison to the correlation of other strains to EDL933 or K12.

**Table 7:** Analysis of microarray hybridisation data from 19 unsequenced test strains. The CGH microarray data was cleaned, normalised and cut-off assessed using the MKD method, then the Pearson's correlation co-efficient was calculated for comparison between each test and reference strain. The highest correlation is shown below. The strain ID, source and slide codes for each of the test strains is included

Strain	Source	Slide replicate numbers			Reference strain most correlated with	
					
					Strain	Pearson Correlation Coefficient
0023/99	Bovine	13248842	12842688		EDL933	0.78
0059/99	Bovine	12842681	13252965		EDL933	0.83
0144/99	Ovine	12842593	13248844		EDL933	0.83
0445/99	Ovine	12842605	12842576		EDL933	0.85
0796/00	Bovine	12842610	12842591		EDL933	0.78
1299/00	Human	12842608	13248843		EDL933	0.83
1463/00	Human	12842620	12842580		EDL933	0.85
1464/00	Human	12842621	12842581		EDL933	0.79
1471/00	Human	13252961	13248846	12842615	EDL933	0.81
1472/00	Human	13252963	12842582	12842614	EDL933	0.86
1484/00	Bovine (Burger)	12842669	13248838		EDL933	0.66
1489/00	Bovine (Steak)	12842668	12842577		EDL933	0.75
1812/00	Bovine	12842666	12842578		EDL933	0.81
1585/00	Bovine	12842603	12842586		EDL933	0.88
0945/00	Bovine	12842682	12842583		EDL933	0.79
0330/01	Bovine	12842680	13248839		EDL933	0.84
0864/00	Bovine	13248841	12842613		EDL933	0.82
1070/00	Bovine	12842678	13248840		Sakai	0.59
1176/00	Bovine	12842601	12842585		MG1655	0.85

The second output of the analyses step identified genes that were consistently present for the K-12 like O157 strain and MG1655, and absent for EDL933 or Sakai like O157 strains. As a result a list of 401 genes that were consistently present for the O157 gene set and consistently absent from the K-12 gene set, was made. Inversely there were 11 genes that were consistently present in the K-12 gene set and consistently absent in the O157 gene set, a list of which was also generated (data not shown). Genes from both lists were collated to form the third output consisting of 412 variant genes that were unique markers for each group. The gene list and the biological significance of these findings are currently being investigated and are likely to give clue to new virulence factors harboured by O157 strains [31].

### Determining the effect of gene copy number and spatial correlation within bacterial genomes

**Figure 5:**
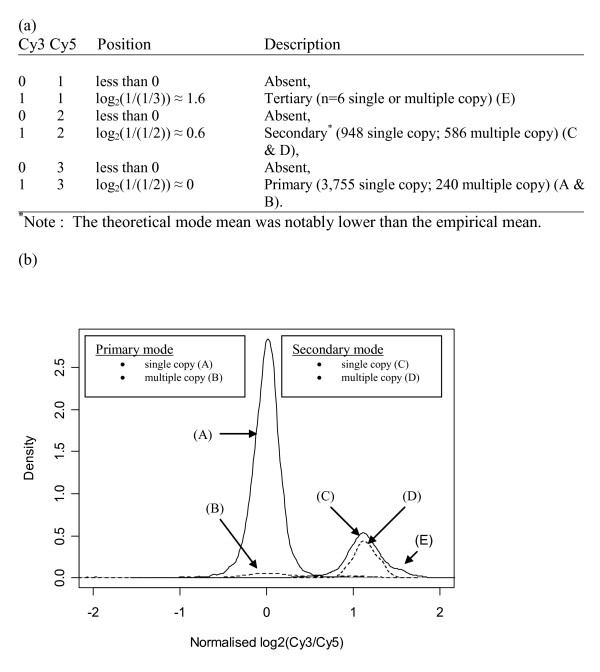
**The empirical density of the Sakai strain partitioned into single and multiple copy genes**. (a) The theoretical location of the four modes, assuming a constant coefficient of hybridisation and labelling. (b) The empirical density of the Sakai data. The primary mode consists of 3,755 and 240 genes present in all three of the sequenced strains on the Cy5 channel highlighted as single and multiple gene copies (solid line and broken line, respectively). The secondary mode includes genes that are specific to only Sakai strain and consists of 948 and 586 genes in single and multiple copies (solid and broken lines, respectively).

To investigate whether there was a copy number effect in our data we compared single copy genes to known multiple copy genes in our control strains to look for differences in the normalised log_2_(Cy3/Cy5) data. To do this we needed to first account for the effect of using pooled reference DNA. In our data set 3,755 genes were present in all three genomes, whereas 948 genes were present in EDL933 and Sakai, 5 genes were present in MG1655 and EDL933. There were 436, 79 and 11 genes unique to MG1655, EDL933 and Sakai, respectively. Furthermore, 826 gene probes had greater than 80% identity with more than one region of the genome, causing a natural shouldering effect. The majority of probes with multiple hits (586 probes) were found in EDL933 and Sakai, and 240 of these were found in all three strains [31]. Figure 5 presents two modes, the primary and secondary mode that resulted when Sakai was used as the test strain. Genes included in the primary mode are present in all three genomes in single and multiple copy (MG1655, EDL933 and Sakai). Whereas the secondary mode include genes present only in EDL933 and Sakai genomes, both in single and multiple copy. Therefore, within these two modes there was no difference in log_2_(Cy3/Cy5) distribution between genes with single and multiple copy numbers throughout the chromosome. Therefore the source of variation in these log_2_(Cy3/Cy5) distributions were not caused by copy number differences within the bacterial genome but instead by the number of copies of each gene present in the control Cy5 channel as a result of using pooled reference DNA.

**Figure 6:**
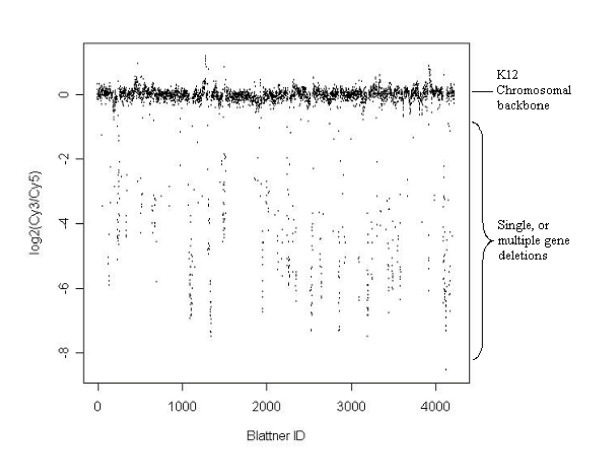
**The Sakai log_2_(Cy3/Cy5) data indexed by the MG1655 genome**. The K12 chromosomal backbone and single and multiple contiguous gene deletions harboured in Sakai with respect to the MG1655 chromosome are shown.

We went on to examine the extent of genomic spatial correlation in bacterial genomes, which has been shown to be important in eukaryotic studies [6-8]. The O157 genome comprises essentially of a K12 chromosomal backbone that is interspersed with regions of insertions and deletions. Therefore we examined the microarray data resulting from the Sakai strain, using genes within the K12 genome. For looking at spatial correlations typical improvements can be gained by modelling smoothed or segmented log_2_(Cy3/Cy5) data. So, we assessed the ratio after smoothing with both an unweighted and a weighted moving average to the log_2_(Cy3/Cy5) data [32](data not shown). Then the MKD algorithm was applied to the moving average scores and sensitivity, specificity and M-scores were generated. The sensitivity and specificity calculated were approximately 75% and 85%, respectively, and the M-score was less than 90%, which is much lower than when the simple moving average adjustment had not been applied (Tables 4 to 6). We believe this is due to many single deletion events occurring in the K12 genome represented in the Sakai strain, with respect to MG1655. Figure 6 shows the Sakai data as indexed by the MG1655 chromosome. Within this plot the extent of the individual and multiple contiguous gene deletions for the Sakai genome can be seen. In case of the single gene deletions the effect of smoothing and segmentation would cause a reduction in performance of the cut-off algorithm rather than offering any improvement.

## Discussion

Using both the sequenced and unsequenced strains, we have generated a process for bacterial CGH microarray data analysis, as shown in Figure 1. Although this process appears intuitive, steps are often left out of analysis plans leading to a mis-interpretation of results [33-35]. Key advantages of the process described here includes a clear and simple process allowing bench scientists access to transparent analysis ideas, as well as to database repository curators through our reliability metric for data inclusion in terms of a minimum M, or sensitivity and specificity scores [29].

A major difference in this study compared to many CGH experiments was using a pooled reference DNA from three sequenced strains where *in silico *information was available (MG1655, EDL933 and Sakai). It was found that the number of strains present in the pooled reference was the major source of variation in signal intensity; as we found no evidence in bacterial genomes that multiple copy genes had any increase of signal intensity ratio compared to single copy genes (Figure 5). This is in contrast to CGH microarray studies used to analyse aberrations in tumour or cancer cells. In such studies a shift would be expected in mean of the multiple copy genes compared to the single copy genes [4,5]. Although our results may be an effect of using multiple genomic DNA in the control channels for our experiments, we believed that even if the array experimental design consisted of only a single genomic DNA in the Cy5 channel this effect would not replicate the eukaryotic tumour examples. The difference may be because within bacteria genomes multiple copy aberrations occur less frequently and to a lesser degree than in eukaryotic tumour cells, but requires further examination. A further difference highlighted in this study between bacterial and eukaryotic CGH microarray studies was the effect of spatial correlation. In this study we found that application of a weighted and unweighted moving average to the log_2_(Cy3/Cy5) scores to account for correlation between adjacent genes [36], in fact decreased the sensitivity diagnostics. However, an adaptation of this method may be more appropriate for bacterial genomes and worth investigating in future.

The use of pooled DNA as control for CGH array experiments is novel and in contrast to current practice in bacterial CGH microarray studies where often only one genome is represented in the control channel, despite the array representing several bacterial genomes. Our method proved that inclusion of genomic DNA from three genomes not only enabled all spots on the array to be validated but also provided greater coverage to understand the bacterial genomic diversity present in EDL933 and Sakai-like strains in comparison to K12-like strains.

As a result this process has identified each sequenced strain most similar to the 19 test strains included in this study, using Pearson correlation coefficient, and a set of genes have been identified that separates the O157 group from K12. The invariant genes within the EDL933 and Sakai subgroup may be indicative of potential virulence markers, whilst the MG1655 (K12) subgroup may be genetic markers present in potentially commensal strains, and is currently being investigated in further detail [31]. The process described in this paper delivers the flow of information through bacterial CGH studies from raw data to the final analysis stage. To date, no software has been made available to fully encapsulate this process to understand the mosaic nature of bacterial genomes.

The process included data cleaning and normalisation within the pre-processing step. The data cleaning step not only ensured the exclusion of control spots but also the removal of data with poor signal intensity, and is crucial to the correct interpretation of the data. Although normalisation is not mandatory, it is highly recommended Common normalisation approaches have included: dividing by the control channel; control genes; global slide mean intensity corrections; print tip median correction; and a loess print tip normalisation method [37-39], a combination of several of these approaches were used in this study (see Materials and Methods).

Various methods have been described promoting ways to differentiate divergent or absent genes from conserved genes within bacterial CGH microarray data sets. In essence, this appears a simple task, and some researchers have used the midway point between conserved and divergent genes [40]. Here, some of the more widely used algorithms to determine the cut-off in bacterial CGH studies were compared, along with two novel approaches, the MKD and mixture modelling approaches.

To compare the algorithms reliability metrics were calculated and contrasted. The algorithms were compared by combining the number of genes to be identified as present and absent or divergent from each cut-off with BLASTN data. An identity of greater than 80% produced by BLASTN search matched with hybridisation signal intensity. The validation step within the process allowed the quantification of correct and false classification of genes for the sequenced strains.

Since, both the reference strains, and unknown strains were carried out from the same suite of experiments, the reliability in the validation stage of Figure 1 was high as shown in Tables 4 to 6. These results from the control sequenced strains provided confidence so it was expected that the test strains would provide similar results.

From the analysis carried out on three separate sequenced strains of *E. coli*, we see that the naïve cut-off is a good approach at partitioning the genes into conserved and divergent, if the empirical evidence is used. However, a range of cut-off values need to be considered for this approach and the cut-off value found most suitable in this study was 0.25 for EDL933 and MG1655, and 0.2 for the Sakai data set. Hence, using a fixed midway cut-off is potentially misleading, since the inherent variability between experiments could result in differences between different slide and isolates, especially as each hybridisation and normalisation will lead to a different distribution of Cy3/Cy5 per slide. Therefore, because the method is not automatic, and the cut-off will vary between array-slides and experiments, the naïve cut-off is not appropriate in an unsupervised manner.

The cut-off between present and absent or divergent genes was defined as the position of presence when using the GENCOM and Prowollik methods. However, the GENCOM, GACK, Porwollik algorithms are bound by the assumptions of symmetry, normality, and linearity, which were reflected in their M-scores. The further away the data is from meeting these assumptions the less able the algorithms were at correctly identifying conserved genes. Therefore these assumptions can be invalidated when using genes that have multiple copies, or hybridise at lower than 100% BLASTN identity match which creates natural shoulders on the primary mode [36]. Additionally, the GENCOM, GACK, Porwollik methods are likely to produce poor results when using a combination of strains for the reference control channel, as has been used in this study, and may be used for other studies where more than one bacterial genome is present on the array.

The similarity between the GENCOM and GACK algorithms has already been reported [41]. In a larger study the GACK was found conservative, since it did not describe genes that are either clearly present, or divergent/absent [42]. Intuitively, a conservative estimate of the number of genes present is appealing. However, this complicated the downstream analysis process by classifying genes as present, absent, or not having enough evidence to decide. It introduced complacency into the analysis since miss-classifications were still present. Furthermore, it may not be helpful since by not classifying the genes, they are either ignored from any downstream analysis, or inflate the pool of those possibly present. Hence, the trade off between sensitivity and specificity should be considered, rather than ignored.

Comparison between the Prowollik and MKD methods presented some differences between the algorithm results. Whereas the mixture modelling algorithm performed well in both the sensitivity and specificity, as shown in Tables 4 to 6. This cut-off algorithm had the advantage of modelling the gene variability rather than just the mean log_2_(Cy3/Cy5) value for each gene. Thus, providing a genuine estimated gene probability of presence. This algorithm did have the disadvantage of modelling an unknown number of modes, suffered convergence complications, and was computationally intensive.

## Conclusion

In summary the results indicated that the MKD method showed good sensitivity and specificity, and could be automated easily in future due to its simplicity. It was found that the time taken, the level of complexity and implementation was a disadvantage for the mixture model, whilst offering little improvement from the simpler MKD algorithm. Also, the interpretation and understanding is more straightforward for the MKD algorithm than the alternatives, and is non-parametric. Therefore, by using the simplest, but most informative algorithm the analysis was more inclusive and directed towards the empirical evidence.

The advent of genome sequencing has brought about a new era in understanding biological processes and has also driven the development of methods such as CGH microarrays to exploit this information. In this study we have described a process that encapsulates all the stages required for analysis of bacterial CGH microarray data, and included a way of ensuring robust conclusions. Although the bacterial CGH experiments described in this paper involve looking at diversity within bacterial genomes, *Escherichia coli *in particular, we believe that the method can be extended to any bacterial CGH microarray study.

## Methods

### Bacterial strains and isolation of genomic DNA

Three *E. coli *sequenced reference strains, MG1655 (K-12), O157:H7 (EDL933), and O157:H7 (Sakai), and 19 *E. coli *O157 test strains were included in this study. The *E. coli *O157 test strains were collected in a previous study from human, animal, and meat sources from different regions in England [31]. The strains were selected based on the maximum diversity in their Pulse Field Gel Electrophoresis profiles and included human and animal isolates.

Genomic DNA was isolated from overnight cultures of bacteria grown aerobically in LB broth. Cultures were boiled for ten minutes before centrifugation and DNA extracted using QIAGEN Dneasy tissue kit (no. 69504; QIAGEN). The microarray experiments were performed in triplicate for the three sequenced strains, *E. coli *MG1655; EDL933; and Sakia and at least in duplicate for the test strains.

### Construction and Microarray hybridisation conditions

The *E. coli *panarray contained 70 base pair oligonucleotide probes (Array-ready Oligo Set v 1.0; Operon) representing 5,978 chromosomal open reading frames (ORFs) from three *E. coli *strains, K-12 (MG1655), O157:H7 (EDL933), and O157:H7 (Sakai) and 110 ORFs from pO157 and pOSAK1 plasmids.

The microarray was printed from oligonucleotide probes dissolved in Proton™ Universal Spotting Solution at a concentration of 40 μM, then spotted on UltraGAPS slides (Corning) with a MicroGrid II microarrayer (Genomic Solutions). The slides were printed in a 4 column by 8 row block design that were replicated twice within slide and were controlled by the TAS application. Each sub-grid consisted of 21 by 21 spots with a 0.21 mm spacing between the spotted targets.

A reference experimental design was implemented. This compared a constant control, to each strain. Within the control channel was DNA from all three sequenced strains, where each strain contributed a third of the total DNA i.e. 0.66 μg. The test channels used DNA from one strain per microarray slide. Two microgram of DNA was used for the labelling, according to the protocol at the Institute of Food Research IFR[43] and 0.5 μl of Cy5 or Cy3 dCTP (1 mM stock, Amersham) was found to be adequate for our purpose. The probe purification and hybridisation was performed according to the protocol developed by BμG@S [44], except that slides were washed twice gently in Wash A (1 × SSC, 0.05% SDS) for at least 5 min each. For each strain, the hybridization experiments were performed at least in duplicate. The processed slides were scanned using a GenePix 4000 B scanner with software GenePix Pro 4.1 (Axon Instruments, Inc). This used the standard adaptive circle segmentation method and converted the information from images into a value between 2^0 ^to 2^16^. The flagging convention was to define a good spot as having a minimal of 65% of its pixels larger than background plus two standard deviations. Both pre- and post-normalised array data are available from Array-Express, experiment number E-MEXP-945 [45].

### Process for the analysis of bacterial CGH data

The five steps in the CGH process were: data cleaning; normalising, cut-off, validation, and analysis. These are explained as follows and shown in Figure 1:

#### 1. Data cleaning

The data cleaning included a median background correction according to the method of Yang *et al*, 2000 [46]. This was followed by quality control checks to remove poor quality hybridisation spots, and control spots (e.g. landing lights, genes from other organisms)[47].

#### 2. Normalisation

We implemented two normalisation steps, these included dividing by the control channel; and correcting for the print tip control gene median, where the print tip relates to hybridisation results from the same block [47]. The control genes comprised of genes known to be present in all 3 reference strains from the BLASTN data. The control gene median was the median value for the control genes present in each print tip. We produced ratio intensity (RI) plots both prior and post normalisation to assess the effect of normalisation and the quality of the hybridisation data.

#### 3. Cut-off algorithms

After normalisation six cut-off algorithms were compared, these included the naïve cut-off; GENCOM; GACK; Porwollik; Minimum Kernel Density; and Mixture modelling methods, as follows.

##### The naive cut-off

In this method a point estimate was imputed to differentiate between conserved and variable genes across all microarray slides. Five cut-off values of 0.5, 0.33, 0.25, 0.2, 0.1 were separately implemented to determine presence or absence of each gene from our data set. This resulted in five presence/absence vectors that were compared to BLASTN data.

##### The GENCOM approach

This is an iterative method, which implemented a simple linear regression between the Cy5 and Cy3 channels on the log_n _scale for the hybridisation data. Here *R*_*p *_and *G*_*p *_denote the mean Cy5 and Cy3 fluorescence signals, respectively, from the present gene set. The initial present set of genes were found by using Equation 1,

ln(*R*_*t*_) <*α*_*p *_+ *β*_*p *_ln(*G*_*t*_) - 3*σ*_*p*_, **or **ln(*R*_*t*_) > *α*_*p *_+ *β*_*p*_. ln(*G*_*t*_) + 3*σ*_*p *_

where *α*_*p*_, *β*_*p*_, and *σ*_*p *_were initially set to 0, 1, and 0.05 respectively and genes were classified as present if they meet either condition. Then, a simple linear regression model was fitted to the present data set to estimate *α*_*p*_, *β *_*p*_, and *σ*_*p*_, which represented the regression intercept, slope and standard error, shown in Equation 2,

ln(*R*_*t*_) <*α*_*p *_+ *β*_*p *_.ln(*G*_*p*_).

Now using the estimates for *α*_*p*_, *β *_*p*_, and *σ*_*p*_, in Equation 1, a larger set of present genes was found, and these were used to re-estimate the parameters. The process was repeated until the present gene set no longer changed from iteration to iteration [48].

##### The GACK approach

This method acknowledges the danger of selecting cut-offs blindly without empirical evaluation, because of inherent differences in variability between technology, and analysis methodology. The algorithm was used to find the location and height of the major peak of the log_2_(Cy3/Cy5) distribution, then values of log_2_(Cy3/Cy5), that represent half the major peak height were determined. Using this subset a normal probability density function was fitted to the peak, and extended to cover the range of the log_2_(Cy3/Cy5) observations. The estimated probability of presence (EPP) provided a level of confidence for observations being reported as present. The EPP was calculated as,

%EPP = 100 × (normal probability function/observed function),

This method relied heavily on the assumptions of symmetry and normality of the log_2_(Cy3/Cy5) distribution [49].

The GACK algorithm was implemented to determine the cut off of the 5,744 genes. Two different EPP values of 0, and 50 were used. The value of EPP assigned determined the confidence in experimental data such that using 0 EPP gave the least confidence whilst 50 EPP gave most confidence.

##### The Porwollik method

This is a two-stage method. Stage one partitions the gene-mean Cy3/Cy5 distribution into initial present/absent gene sets. Hence, genes with an average Cy3/Cy5 < 0.5 were assumed absent and included in gene-set *x*, those genes with an average Cy3/Cy5 > 0.65 were assumed present and included in gene-set *y*.

After calculating the present and absent set means and standard deviations, the presence/absence of each gene was re-evaluated using,

$$\begin{array}{ll} \bar{x}+2.s_{x}>\log_{2}(Cy3/Cy5) & \text{(absent gene-set)}\\ \bar{y}-2.s_{y}<\log_{2}(Cy3/Cy5) & \text{(present gene-set)} \end{array}$$

where $\bar{x}$, and $\bar{y}$, were the absent and present gene-means; and s_x_, s_y_, were the absent, and present gene subset standard deviations. This is a further method that relies on symmetry and normality of the Cy3/Cy5 distribution [19].

##### The Minimum Kernel Density cut-off (MKD)

In this method a kernel density estimator was fitted to the log_2_(Cy3/Cy5) distribution to determine the minimum value between the conserved and divergent regions. The method is analogous to smoothing the log_2_(Cy3/Cy5) distribution and using the minimum to bisect the present and absent regions. The kernel density is shown below as,

$$\hat{f}(x)=\frac{1}{n}\sum_{i=1}^{n}K\left(\frac{x-x_{i}}{h}\right),$$

where *K *is the kernel function, most usually a Gaussian, *x*_*i *_is the gene-mean, *n *is the number of genes, *h *is the bandwidth, and the standard deviation of the function. Although this method uses a Gaussian kernel, it makes no assumptions of normality [50]. The method was carried out on data from each hybridisation in our data set, and then strain replicates were summarized.

##### The Mixture Modelling approach

This fits a mixture of normal distributions to the empirical data dynamically using a variance component model. Hence, the genes were summarised, and a probability calculated, by comparing the actual log_2_(Cy3/Cy5) value to function and assuming the gene is truly present. In the case of a mixture of two normal distributions, the log likelihood can be shown as,

$$LogL(\psi ;x_{ij})=\sum_{i=1}^{n}\log_{n}\left(\alpha .f(\mu_{1},\sigma_{\mu 1}^{2},\sigma_{\varepsilon }^{2};x_{ij})+(1-\alpha ).f(\mu_{2},\sigma_{\mu 2}^{2},\sigma_{\varepsilon }^{2};x_{ij})\right);$$

where *ψ *= (*α*, *μ*_1_, *μ*_2_, *σ*_*μ*1_^2^, *σ*_*μ*2_^2^, *σ*_*ε*_^2^), *x*_*ij *_are the log_2_(Cy3/Cy5) values for the *i*^*th *^gene, and *j*^*th *^replicate; *α *is the proportion of genes estimated as absent; *μ*_1 _is the mean log_2_(Cy3/Cy5) value calculated from the absent genes;*μ*_2 _is the mean log_2_(Cy3/Cy5) value for the present genes; *σ*_*μ*1_^2 ^is the between-gene variance for the absent genes; *σ*_*μ*2_^2 ^is the between-gene variance for the present genes, and *σ*_*ε*_^2 ^is the within-gene variance. The function *f*, can be shown as Equation 5,

$$f(\mu_{k},\sigma_{\mu k}^{2},\sigma_{\varepsilon }^{2};x_{ij})=\frac{\exp^{\left[-\frac{\left(n_{i}-1\right)s_{i}^{2}}{2\sigma_{\varepsilon }^{2}}-\frac{{\left({\bar{x}}_{i}-\mu_{k}\right)}^{2}}{2(\sigma_{\mu j}^{2}+\sigma_{\varepsilon }^{2}/n_{i})}\right]}}{{\left(2\pi \right)}^{n_{i}/2}\sigma_{\varepsilon }^{2}(\frac{n_{i}-1}{2})n_{i}^{\frac{1}{2}}{\left(\sigma_{\mu k}^{2}+\sigma_{\varepsilon }^{2}/n_{i}\right)}^{1/2}},$$

where ${\bar{x}}_{i}$ is the *i*^*th *^gene-mean, $s_{i}^{2}$ is the *i*^*th *^within gene-sample variance, and *k *is the distribution (absent = 1, and present, or conserved = 2). When many genes have multiple copies, a greater number of distributions may be preferred this could significantly reduce the variances of the parameter estimates [51-54].

#### 4. Validation

Figure 1 shows the validation process that was performed in parallel to analysis. To validate the microarray process the true presence of the unique region of each gene, represented by the oligonucleotide probe, was searched by BLASTN at  against the fully sequenced genomes of MG1655, EDL933 and Sakai. These data were compared against the microarray hybridisation results after applying each cut-off algorithm to gauge an appreciation of how well the process was performing. The sensitivity and specificity were calculated to summarise the reliability of the results found. These summaries are called the sensitivity, specificity, and they were combined using the M-Score, weighted by prevalence [24]. We have used the M-Score as a single combined metric to rank the reliability of the two statistics.

#### 5. Analysis

After the reference strains were found to be highly sensitive and specific, three analysis steps were carried out for the test strains:

a) Correlation coefficients were computed between reference and test strains. The test strains were labelled as similar to the reference strain with the highest correlation and formed two groups.

b) Within each of the generic groups, genes were categorised as: consistently absent; consistently present; or mixed.

c) Genes that were consistent within a generic group, but inconsistent between groups were used to define the difference in biological characteristic between each group.

## Determining the effect of gene copy number

To assess the effect of copy number within the genome we used *in silico *data to study the theoretical log_2_(Cy3/Cy5) value. Then we compared the single verses multiple gene copy distributions.

## Spatial correlation

To consider the effect of serial correlation between genes of the same orientation a moving average was used to smooth data closely positioned on the chromosome [9]. This was implemented with a three point moving average across the genome with the centre point being equally and double weighted at the centre position. Validation metrics were then compared with and without the smoothing applied to the log_2_(Cy3/Cy5) data.

## Authors' contributions

MW and MA conceived and were successfully funded for this study. GU obtained the strains, carried out the molecular work and reported exploratory findings. BC reviewed the literature, established the statistical methodology and validated the array platform. BC and MA drafted the paper. All authors read and approved the final manuscript.
